# Retrospective analysis of sorafenib combined with interferon α-1b, interleukin-2, and thalidomide as maintenance therapy in FLT3-ITD-positive acute myeloid leukemia

**DOI:** 10.3389/fonc.2025.1698935

**Published:** 2025-11-12

**Authors:** Cheng Cheng, Ruihua Mi, Dongbei Li, Lin Chen, Xudong Wei

**Affiliations:** 1 Department of Hematology, The Affiliated Cancer Hospital of Zhengzhou University & Henan Cancer Hospital, Zhengzhou, China; 2 Central Laboratory, The Affiliated Cancer Hospital of Zhengzhou University & Henan Cancer Hospital, Zhengzhou, Henan, China; 3 Institute of Cancer Research, Henan Academy of Innovations in Medical Science, Zhengzhou, China

**Keywords:** sorafenib, interferon α-1b, interleukin-2, thalidomide, FLT3-ITD, acute myeloid leukemia

## Abstract

**Objective:**

To evaluate the clinical efficacy of maintenance therapy with sorafenib combined with interferon α-1b, interleukin-2, and thalidomide (the ITI regimen) in patients with FLT3-ITD-positive acute myeloid leukemia (AML).

**Methods:**

19 FLT3-ITD(+) AML patients were retrospectively analyzed, who received the sorafenib combined with ITI regimen as maintenance therapy after achieving remission at Affiliated Cancer Hospital of Zhengzhou University (January 2014–December 2024). Minimal residual disease (MRD) levels were monitored, and clinical outcomes, including survival duration, were assessed.

**Results:**

This study included 19 patients (9 males, 10 females) with a median age at diagnosis of 59 years (range: 21–76). The median white blood cell count was 32.25×10^9^/L (range: 0.7–254×10^9^/L). Among them, 13 patients (68.4%) maintained sustained MRD negativity during sorafenib combined with TI regimen therapy, while 3 patients experienced morphological relapse and 3 had molecular relapse. Median overall survival (mOS) was not reached, with 12- and 24-month OS rates of 100% (19/19) and 94.7% (18/19), respectively. During maintenance therapy with sorafenib combined with ITI, median relapse-free survival (mRFS) was also not reached, with 12- and 24-month RFS rates of 73.7% (14/19) and 57.9% (11/19), respectively.

**Conclusion:**

The sorafenib combined with ITI regimen is an effective maintenance therapy for FLT3-ITD (+) AML, significantly reducing relapse risk and prolonging survival.

## Introduction

Acute myeloid leukemia (AML) is a highly heterogeneous hematologic malignancy. Allogeneic hematopoietic stem cell transplantation (allo-HSCT) remains the optimal curative approach for intermediate- and adverse-risk AML patients (1, 2). However, for transplant-ineligible patients, effective minimal residual disease (MRD)-directed interventions to prevent relapse during post-remission therapy represent a critical unmet clinical need (3). Approximately 30% of AML patients harbor FMS-like tyrosine kinase 3 (FLT3) mutations, which are associated with high blast counts, low complete remission (CR) rates, reduced disease-free survival and overall survival (OS) (4, 5). Sorafenib, a small-molecule tyrosine kinase inhibitor targeting FLT3, exerts its antileukemic effects by competitively binding to the ATP-binding site, thereby inhibiting kinase activity, disrupting downstream signaling, and promoting leukemic cell differentiation and apoptosis (6–8). Multiple studies have demonstrated the efficacy of sorafenib in FLT3-ITD-mutated AML (9, 10). Our team previously reported promising results with the interferon-α1b, interleukin-2, and thalidomide (ITI) regimen, both in refractory/relapsed AML and MRD-positive AML (11, 12).

This study focuses on transplant-ineligible, FLT3-ITD(+) AML patients in remission post-chemotherapy, evaluating the combination of sorafenib and the ITI regimen as maintenance therapy, with close monitoring of MRD dynamics and survival outcomes, aiming to provide novel clinical insights and therapeutic strategies.

## Materials and methods

### Patients

19 FLT3-ITD-mutated AML patients treated at the Affiliated Cancer Hospital of Zhengzhou University (January 2014–December 2024). Diagnosis was confirmed based on clinical presentation, bone marrow morphology, flow cytometric immunophenotyping, cytogenetics, and molecular profiling. The sorafenib combined with ITI regimen was initiated as maintenance therapy upon completion of all planned consolidation chemotherapy cycles, while patients were in morphological CR. The patients were considered not to be candidates for allo-HSCT primarily due to (1): patient preference or financial reasons (2); significant comorbidities and/or poor performance status; or (3) lack of a suitable donor. The diagnostic criteria for AML were based on the relevant standards established by the World Health Organization (WHO) in 2016, while the classification followed the FAB (French-American-British) classification system. The patient’s risk stratification was assessed according to the European Leukemia Net (ELN) 2022 risk classification for acute myeloid leukemia. This study was approved by the Ethics Committee of Henan Cancer Hospital (No: 2022-548-001). This study adhered to the tenets of the Declaration of Helsinki. Informed consent was obtained from all guardians or patients.

### Treatment protocol

Patients received sorafenib (400 mg orally twice daily) combined with the ITI regimen consisting of subcutaneous interferon α-1b (60 μg every other day), interleukin-2 (1 million IU every other day), and oral thalidomide (100 mg nightly) in 28-day cycles. For thromboprophylaxis in patients with platelet counts >50×10^9^/L, compound Danshen tablets (3 tablets three times daily) were administered. Preemptive ibuprofen was administered orally 1 hour before interferon α-1b injections to prevent treatment-related fever.

### Treatment response monitoring

Patients underwent monthly bone marrow evaluations during sorafenib plus ITI regimen therapy, including morphologic assessment (complete remission defined as <5% blasts) and MRD monitoring. MRD was assessed using EDTA-anticoagulated samples for quantitative PCR (fusion gene-positive cases) and heparinized samples for 10-color flow cytometry (FCM), with positivity defined as ≥ 0.01% blast cells. The FCM analysis utilized two comprehensive antibody panels (CD34/CD117/CD13/CD38/CD15/CD33/HLA-DR/CD45 and CD34/CD117/CD7/CD56/CD64/CD19/CD45) with ≥ 500, 000 acquired events. For patients with fusion genes (AML1-ETO), RNA was extracted from bone marrow samples and analyzed by real-time PCR using an ABI Prism 7500 system (Applied Biosystems; Foster City, CA, USA), with ABL as the endogenous control. The AML1-ETO assay employed specific primers. This sensitive molecular monitoring complemented the multiparameter flow cytometry approach, providing comprehensive MRD assessment.

Forward Primer: 5′-CACCTACCACAGAGCCATCAAA-3′;

Reverse Primer: 5′-ATCCACAGGTGAGTCTGGCATT-3′.

### Safety profile assessment

Safety was evaluated each time before and after the treatment of each cycle. According to the criteria for adverse reaction assessment established by the World Health Organization, adverse reactions were graded from I to IV.

### Follow-up

Patient follow-up was conducted through medical records and telephone interviews until May 31, 2025. OS was calculated from diagnosis to death or last follow-up, while relapse-free survival (RFS) was measured from sorafenib/ITI initiation to relapse (defined as bone marrow blasts >5% or MRD positivity [≥0.01%]) or last contact.

### Statistical analysis

Statistical analysis was performed using SPSS software (version 26.0; IBM Corp., Armonk, NY, USA). Continuous data are presented as median (range) and compared using the Mann-Whitney U test. Categorical variables between groups were compared using the Chi-square test. OS and RFS were analyzed with the Kaplan-Meier method, and survival curves were generated.

## Results

### Patient characteristics

This retrospective study enrolled 19 consecutive FLT3-ITD-positive AML patients (9 males and 10 females) with a median age of 59 years (range: 21-76) at diagnosis. The cohort demonstrated a median presenting white white cell count of 32.25×10^9^/L (range: 0.7-254×10^9^/L). By FAB classification, cases included M1 (n=3, 15.8%), M2 (n=7, 36.8%), M4 (n=3, 15.8%), M5 (n=5, 26.3%), and M7 (n=1, 5.3%). According to the European LeukemiaNet (ELN) 2022 risk stratification criteria for acute myeloid leukemia, patients were categorized as follows: favorable risk in 3 cases (15.8%), intermediate risk in 9 cases (47.4%), and adverse risk in 7 cases (36.8%). All patients achieved complete morphological remission after ≤2 induction cycles. Fourteen patients (73.7%) received sorafenib (400mg twice daily) during both induction and consolidation phases, while 5 (26.3%) initiated sorafenib during consolidation only. Baseline patient characteristics are summarized in **Table 1**. Treatment details, including regimens prior to maintenance and MRD status immediately before initiating the sorafenib-ITI regimen, are detailed in **Table 2**.

**Table 1:** Initial clinical data of 19 AML patients who received intervention therapy with Sorafenib and ITI regimen.

No.	Sex	Age	WBC(10^9/L)	Blast cell (%)	FAB classification	Cytogenetics	Gene mutation(exclude FLT3-ITD)	Fusion gene	Risk stratification
1	M	59	59	81	M2a	Normal	IDH2, NPM1	negative	intermediate
2	F	62	111.58	86	M1	Complex Karyotype	CEBPA, NPM1	negative	adverse
3	M	74	66.4	89	M5a	Normal	NPM1	negative	intermediate
4	F	70	32.25	87.8	M1	Normal	NPM1, CSF3R, IDH2, TET2	negative	intermediate
5	M	61	46.12	75.5	M2b	t (8,21)(q22;q22)	–	AML1-ETO	Favorable
6	M	60	0.7	85.6	M2a	Normal	IDH2, TET2, NPM1, RUNX1	negative	adverse
7	M	60	254	68.8	M5	Normal	IKZF1, TET2, ASXL1	negative	adverse
8	M	47	2.51	34	M2b	t (8,21)(q22;q22)	–	AML1-ETO	Favorable
9	F	43	0.86	76.4	M7	Normal	NPM1, ASXL1	negative	adverse
10	M	21	9.67	87.4	M2b	t(8;21)(q22;q22)	TET2, IKZF1	AML1-ETO	Favorable
11	F	47	40.45	41.6	M5	Normal	TP53, DNMT3A, IDH2, NPM1	negative	adverse
12	F	46	27.83	86.7	M2	Normal	NPM1, IDH2, ABCB1, CYP2C19	negative	intermediate
13	F	55	7.3	52.4	M5	Normal	NPM1, DNMT3A, ABCB1	negative	intermediate
14	F	69	1.26	50	M4	Normal	NPM1, RUNX1, ABCB1	negative	adverse
15	F	76	36.82	95	M1	Normal	NPM1, TET2, CEBPA	negative	intermediate
16	M	70	5.32	53.87	M4	Normal	NPM1, DNMT3A, SMC3, ABCB1	negative	intermediate
17	M	49	47.4	70.8	M2	Complex Karyotype	NPM1	negative	adverse
18	F	37	16.83	75.2	M4	Normal	ABCB1, TET2, NPM1	negative	intermediate
19	F	50	109.1	85.0	M5	Normal	DNMT3A	negative	intermediate

**Table 2:** Pretreatment regimens, treatment responses, and survival outcomes in 19 AML patients.

No.	Timing of sorafenib administration​	Pretreatment regimens	MRD^1^	Status	Relapse-free survival(m)^2^	Overall survival(m)^3^	Outcomes
1	Induction	DA*2, HA, MA, AA, ID-Ara-C*2	negative	CR	72	82	Survival
2	Consolidation	DA, ID-Ara-C, IA, IDA+ID-Ara-C*2, DCAG	negative	molecular relapse	13	76	Survival
3	Induction	CHAG*2, IA.ID-Ara-C, CHAG	negative	CR	126	133	Survival
4	Induction	DCAG*3, ID-Ara-C*2	negative	CR	78	87	Survival
5	Induction	IA*2, ID-Ara-C*3	negative; AML-ETO/ABL 0.0011%	molecular relapse	6	54	Lost to follow-up
6	Induction	IA, ID-Ara-C*3, HA, AA, CAG	negative	morphological relapse	31	41	Death
7	Induction	IA, ID-Ara-C, CHAG, EA, HA	0.06%	morphological relapse	1	21	Lost to follow-up
8	Induction	IA, ID-Ara-C*2, HA, ID-Ara-C*1, HAG, CAG	negative; AML-ETO/ABL 0.0062%	CR	11	24	Survival
9	Induction	CHAG*2	8.85%	morphological relapse	16	20	Death
10	Consolidation	IA, HD-Ara-C, TKI+CAG, CHAG*2, MA, DCHAG*2, HD-Ara-C	negative; AML-ETO/ABL 0.056%	molecular relapse	16	47	Lost to follow-up
11	Consolidation	IA, HD-Ara-C, MD-Ara-C	negative	CR	1	13	Lost to follow-up
12	Induction	IA, HD-Ara-C	negative	CR	38	51	Survival
13	Consolidation	IA, ID-Ara-C*2, HA	0.10%	CR	63	69	Survival
14	Induction	VAH*2, VA*4	negative	CR	1	46	Survival
15	Induction	D-CHAG, Ara-C, DHA	negative	CR	46	50	Survival
16	Induction	VA*9	negative	CR	46	61	Survival
17	Induction	IA, HD-Ara-C*4, AA	2.53%	CR	1	44	Death
18	Consolidation	IA, ID-Ara-C*2	negative	CR	4	59	Survival
19	Induction	IA, ID-Ara-C*2	negative	CR	29	34	Survival

^1^The minimal residual disease (MRD) status of 19 AML patients before receiving sorafenib combined with the ITI regimen.

^2^Relapse-free survival (RFS) was measured from sorafenib/ITI initiation to relapse (defined as bone marrow blasts >5% or MRD positivity [≥0.01%]) or last contact. ^3^Overall Survival was calculated from diagnosis to death or last follow-up,

D, Daunorubicin; A, Cytarabine (Ara-C); H, Homoharringtonine; M, Mitoxantrone; ID/MD/HD-Ara-C, Intermediate/Medium/High-Dose Cytarabine; IDA, Idarubicin; DCAG, Decitabine, Cytarabine, Aclarubicin, and Granulocyte Colony-Stimulating Factor (G-CSF); EA, Etoposide + Cytarabine; VA, Venetoclax + Azacytidine.

### Treatment outcomes

**Figure 1:**
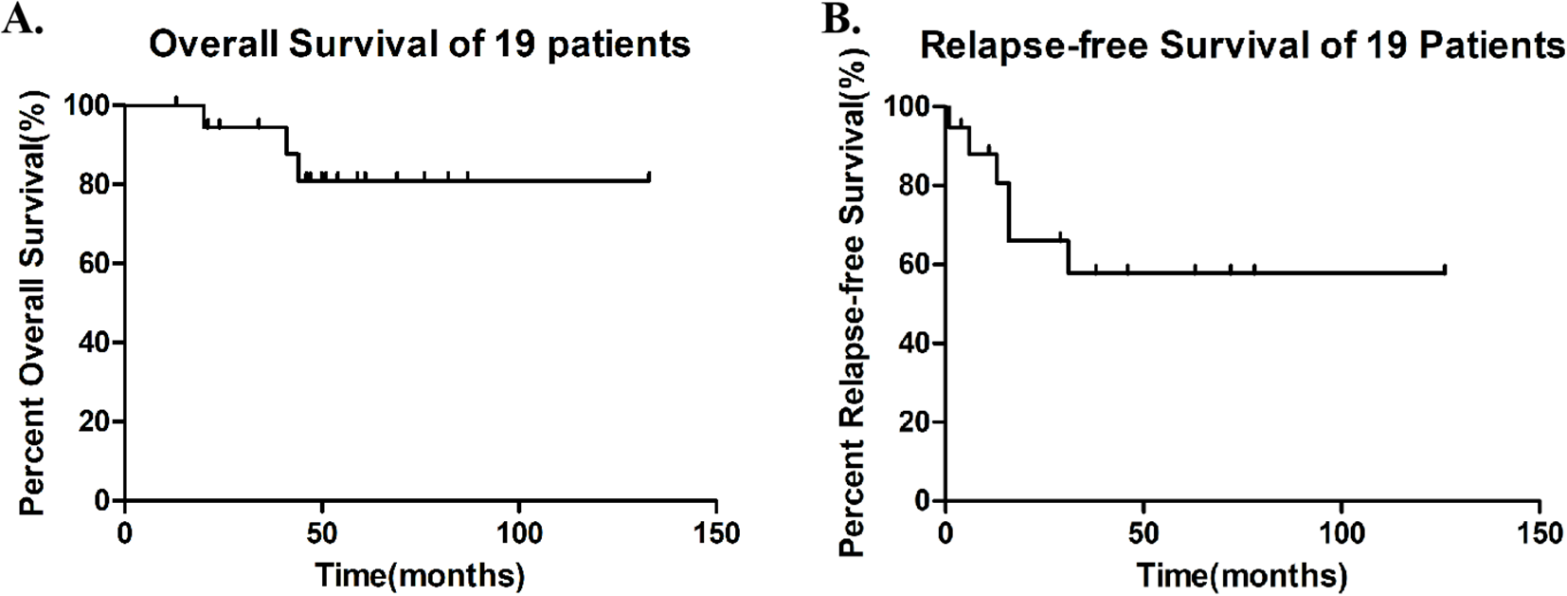
Survival outcomes in 19 patients **(A)** Overall Survival (OS), median OS was not reached, with 12- and 24-month OS rates of 100% and 94.7%. **(B)** Relapse-Free Survival (RFS) during Sorafenib-ITI maintenance, median RFS was not reached, with 12- and 24-month RFS rates of 73.7% and 57.9%.

During maintenance therapy with sorafenib combined with ITI regimen, 13 of 19 patients (68.4%) maintained sustained MRD negativity, while 3 (15.8%) developed molecular relapse and 3 (15.8%) experienced morphological relapse. Notably, among the four patients who were MRD-positive prior to maintenance therapy, Patient 13 achieved MRD negativity after one cycle of treatment and has maintained negative status to date. Patient 9 exhibited a progressive decrease in MRD levels during maintenance therapy and eventually achieved sustained negativity for over five months. Three patients (15.8%), who became eligible for allogeneic HSCT due to the subsequent availability of a matched donor and improved performance status, underwent the procedure after a median of one treatment cycle (range: 1-4) while in CR, despite initial ineligibility from donor unavailability and poor condition post-induction (**Supplementary Table 1**). With a median follow-up of 50 months (range: 13-133), median overall survival (mOS) was not reached (3 deaths, 15.8%), with 12- and 24-month OS rates of 100% and 94.7%, respectively. Similarly, median relapse-free survival (mRFS) was not reached (6 relapses, 31.6%), demonstrating 12- and 24-month RFS rates of 73.7% and 57.9% (**Figure 1**).

### Safety profile and adverse events

The sorafenib and ITI combination regimen exhibited characteristic yet manageable toxicities during maintenance therapy. Hematologic adverse events consisted primarily of grade I–II myelosuppression. Non-hematologic toxicities were predominantly interferon-related constitutional symptoms. Fever occurred in 89.5% of patients (Grade I–II: 84.2%; Grade III: 5.3%), typically lasting 1–3 days after interferon administration. Rhinorrhea (47.4%, all Grade I–II) and myalgia (31.6%, all Grade I–II) were also common, generally resolving within one week. Sorafenib-associated toxicities primarily involved the skin and gastrointestinal tract, including hand-foot skin reaction (36.8%, all Grade I–II), and diarrhea (31.6%, all Grade I–II). All observed toxicities were effectively managed with supportive measures and did not necessitate treatment interruption. The safety profile was consistent with the known effects of each individual agent, and no unexpected toxicities emerged with the combination. No grade IV adverse events or treatment-related mortality were observed.

## Discussion

Current international guidelines, including NCCN and Chinese consensus for adult AML management, uniformly recognize FLT3-ITD mutations as an independent adverse prognostic factor (4, 5, 13). These genetically defined cases exhibit characteristic clinical presentations featuring hyperleukocytosis and elevated tumor burden at diagnosis. Depalmitoylation specifically modulates the biological function of the oncogenic FLT3-ITD mutant, driveing leukemogenesis by activating pathways including PI3K/AKT and RAS/MAPK signaling. This confers intrinsic chemoresistance to patients, manifested as lower complete remission rates and higher relapse incidence (4, 14). The therapeutic challenge is particularly pronounced in elderly patients (≥60 years), where FLT3-ITD co-occurs with age-related biological adversities: impaired organ function, frequent comorbidities, and heightened treatment-related mortality (15, 16). Consequently, only 20-30% of these patients qualify for allogeneic transplantation, the sole potentially curative option. This therapeutic dilemma underscores the critical need for novel maintenance strategies that can prolong remission duration and improve survival outcomes in transplant-ineligible patients while maintaining acceptable toxicity profiles.

Sorafenib, a small-molecule tyrosine kinase inhibitor, inhibits the autophosphorylation of FLT3, as well as the phosphorylation of extracellular signal-regulated kinases 1/2 (ERK1/2) and signal transducer and activator of transcription 5 (Stat5) (17). Meanwhile, sorafenib synergizes with T cells by downregulating ATF4 expression, leading to the activation of the IRF7–IL-15 axis in leukemia cells. This activation subsequently induces metabolic reprogramming of leukemia-reactive T cells *in vivo* (18). Thus making it applicable for targeted therapy of AML. In the SORMAIN study, after a median follow-up of 41.8 months, the median RFS was not reached in the sorafenib group, whereas it was 30.9 months in the placebo group, representing a 75% reduction in the risk of relapse or death (9). In another phase III trial, the 2-year leukemia-free survival rate in the sorafenib group was 78.9%, which was higher than that in the placebo group (56.6%) (19). Based on the evidence that sorafenib as post-transplant maintenance therapy for FLT3-mutated AML can improve relapse-free survival and demonstrate good tolerability, the Acute Leukemia Working Party (ALWP) of the European Society for Blood and Marrow Transplantation (EBMT) recommends using sorafenib to optimize long-term disease control.

Thalidomide, rhIFNα-1b, and IL-2 all exhibit antileukemic activities. Our basic research has found that thalidomide can concentration-dependently inhibit the proliferation of the AML cell line Kasumi-1, and when combined with interferon, it can synergistically suppress cell proliferation and induce apoptosis in this cell line (20). As a lymphokine, IL-2 can promote the proliferation and activation of cytotoxic T cells, natural killer (NK) cells, and lymphokine-activated killer (LAK) cells. It also enhances antibody and IFN secretion by lymphocytes, exerting antiviral, antitumor, and immune-enhancing effects. In patients with core binding factor acute myeloid leukemia (CBF-AML), positive clinical outcomes were evidenced with IL-2 maintenance therapy. Moreover, the histamine dihydrochloride plus low-dose IL-2 regimen confers reduced relapse risk during the post-remission phase of AML through dual mechanisms (1): counteracting myeloid-derived immunosuppression (HDC component), and (2) activating anti-leukemic lymphocytes (including natural killer cells and cytotoxic T lymphocytes; IL-2 component) (21, 22). The three agents demonstrate synergistic effects in antitumor activity and immune regulation, which may make the combination of thalidomide, rhIFNα-1b, and IL-2 a novel effective therapeutic regimen for AML. Our clinical study has shown that the ITI regimen can reduce the level of MRD in AML patients and decrease the risk of relapse. Within our cohort, 20 patients achieving hematologic remission with persistent AML1-ETO fusion positivity received ITI regimen. The fusion transcript became undetectable or decreased in 18 patients, yielding an overall response rate of 90%. Concurrently, we initiated the regimen in 37 MRD-negative patients who had completed all planned consolidation chemotherapy without undergoing allo-HSCT). Maintenance therapy completion rates of 1, 2, 3 year were 75.7%, 59.5%, 40.5% (23). This strategy significantly reduced relapse rates in the treated cohort.

This study evaluated the efficacy of sorafenib combined with the ITI regimen as maintenance therapy in non-transplant eligible FLT3-ITD–positive AML patients in remission after chemotherapy. Among the 19 patients who received this combination, 13 maintained persistent MRD negativity. The longest follow-up was 133 months, and outcomes were encouraging. Additionally, we applied this regimen in three relapsed/refractory AML patients, observing certain therapeutic effects. Our patient cohort encompassed a wide age spectrum, which inherently introduced heterogeneity in the intensity of consolidation chemotherapy administered prior to maintenance, as clinical decisions are guided by individual tolerance and comorbidities. This variation in pre-maintenance treatment intensity is a potential confounding factor in our retrospective analysis. However, the consistent treatment benefit observed, particularly in older individuals often ineligible for intensive chemotherapy, underscores the potential of the sorafenib-ITI regimen. While this remains a study limitation, it also highlights the regimen’s potential applicability across a broad patient population. These findings suggest that this strategy may provide long-term survival benefits, though larger sample sizes and longer follow-up are needed for confirmation, along with mechanistic studies to elucidate underlying processes.

In conclusion, for non-transplant eligible intermediate- to adverse-risk FLT3-ITD–positive AML patients, maintenance therapy with sorafenib combined with the ITI regimen during post-chemotherapy remission effectively suppresses MRD conversion, prevents relapse, prolongs survival, and is associated with minimal adverse reactions and good tolerability. This approach warrants broader clinical application and promotion.

## Data Availability

The original contributions presented in the study are included in the article/**Supplementary Material**. Further inquiries can be directed to the corresponding author.
