# Supplementary material for: Retrospective analysis of sorafenib combined with interferon α-1b, interleukin-2, and thalidomide as maintenance therapy in FLT3-ITD-positive acute myeloid leukemia
Source: Front Oncol. 2025 Nov 12;15:1698935. doi: 10.3389/fonc.2025.1698935 (PMC12646904; doi:10.3389/fonc.2025.1698935)
Supplement: Supplementary file 1 [file Table1.docx]

**Supplementary Table 1.** Clinical Characteristics and Outcomes of the Three FLT3-ITD-Positive AML Patients Who Underwent allo-HSCT

| Patient | Sex | Age (years) | Pre-HSCT Status | Pre-HSCT MRD | Donor Source & Match | Post-HSCT MRD | Post-HSCT FLT3 Mutation | Continued Use of Sorafenib + ITI | Overall Survival (months)¹ |
| --- | --- | --- | --- | --- | --- | --- | --- | --- | --- |
| 1 | F | 47 | CR | Negative | Younger Brother, 10/10 | Negative | Negative | No | 13 |
| 2 | M | 49 | CR | Negative | Daughter, 9/10 | Negative | Negative | No | 44 |
| 3 | F | 37 | CR | Negative | Unrelated Donor, 10/10 | Negative | Negative | No | 59 |

^1^Overall Survival was calculated from diagnosis to death or last follow-up.

**Abbreviations:** allo-HSCT, allogeneic hematopoietic stem cell transplantation; CR, complete remission; MRD, minimal residual disease.
